# Supplementary material for: Proteomics of Paracoccidioides lutzii: Overview of Changes Triggered by Nitrogen Catabolite Repression
Source: J Fungi (Basel). 2023 Nov 12;9(11):1102. doi: 10.3390/jof9111102 (PMC10672198; doi:10.3390/jof9111102)
Supplement: Supplementary file 1 [file jof-09-01102-s001.zip › Supplementary Files.pdf]

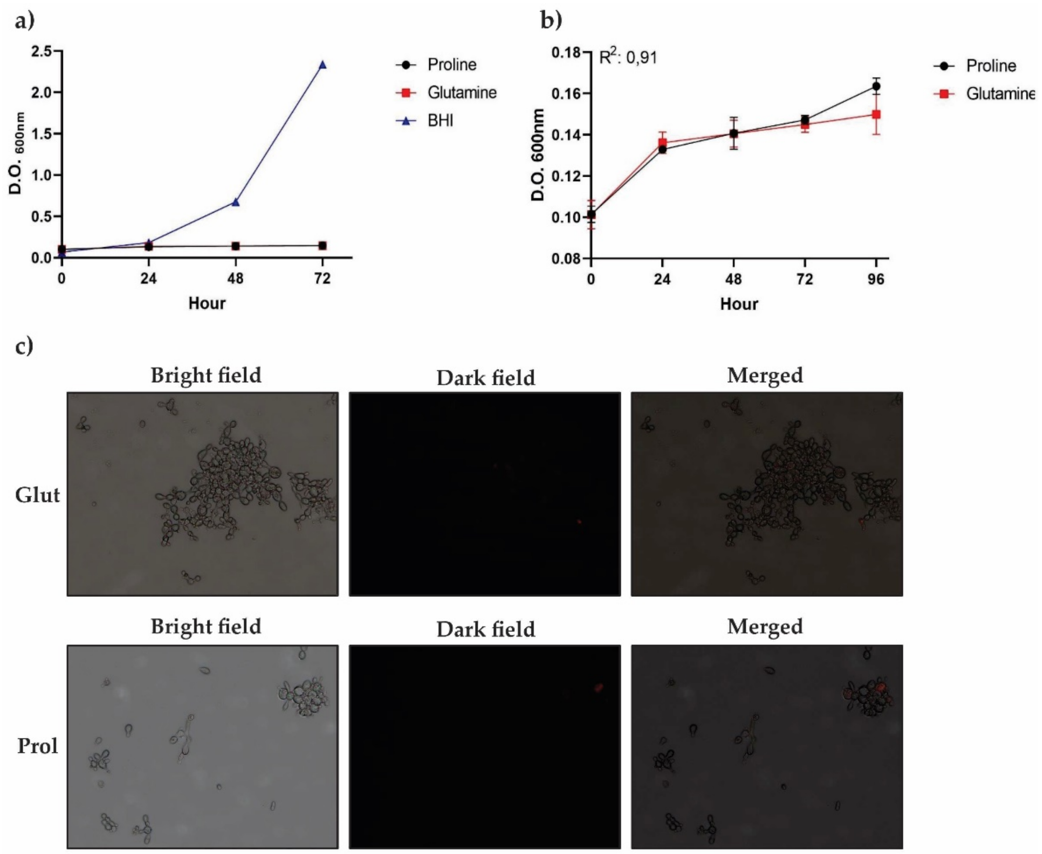

Figure S1. Viability of *P. lutzii* under NCR-conditions. (**a**) *P. lutzii* viability under BHI medium and NCR conditions, supplemented with 10mM of proline and 10 mM of glutamine. (**b**) Viability comparative of *P. lutzii* under NCR conditions. Data are expressed as the mean ± standard deviation of the triplicate of independent experiments ($p \leq 0.05$). Statistical comparisons were performed using Pearson correlation. **(c)** Viability assays were conducted at concentrations of 10 mM proline/or glutamine under NCR conditions. The frames were separated into upper (Glut) and lower (Prol), demonstrating the bright field (light microscopy), dark field (propidium iodide) and merged field (union between the dark and light frame). Non-viable *P. lutzii* yeast cells were stained with propidium iodide (1 μg/mL). Data are expressed as mean ± standard deviation of triplicates of independent experiments ($p \leq 0.05$). Statistical comparisons were performed using Pearson correlation. Prol: proline; Glut: glutamine. Magnification 40x.

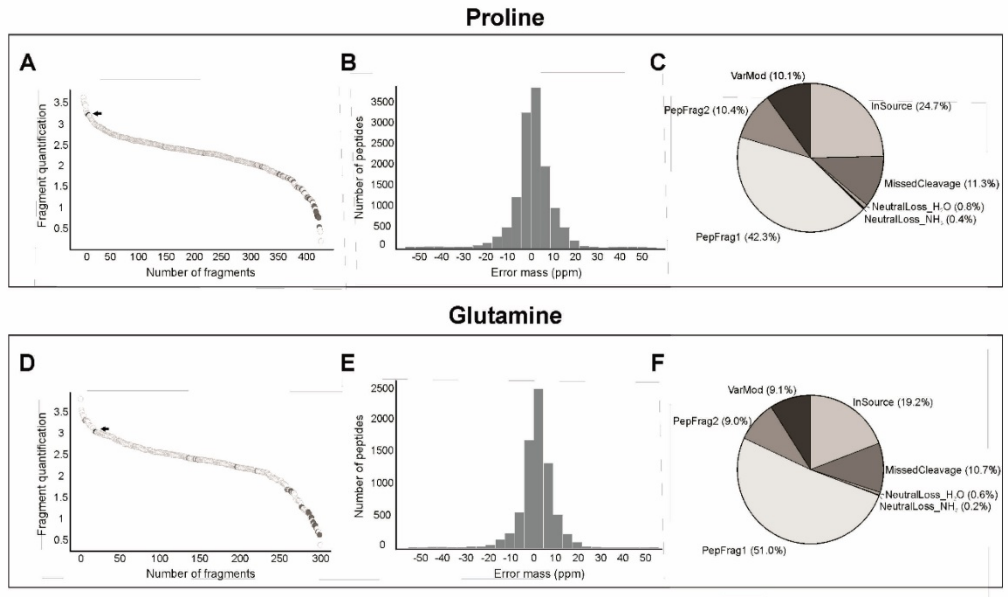

Figure S2. Quality analysis of *P. lutzii* proline and glutamine proteomic data. (**a**) and (**d**) - Dynamic Range. Protein data of each condition was used to calculate the order of magnitude of protein identification and determine the dynamic range. Regular, reverse and standard proteins are represented in blank, grey and black (arrow), respectively. Regular and reverse proteins indicate identified proteins using regular and reverse *P. lutzii* proteome database. The standard protein exhibited an acceptable quantification in both conditions. (**b**) and (**e**) - Peptide Mass Accuracy analyses. Peptide data was used to generate the bar graph showing the mass accuracy for peptides of each condition. The majority of peptides were detected with an error of less than 10 ppm in both proline and glutamine conditions. (**c**) and (**f**) - Peptide detection type. The pie graph brings the percentage of peptides matched against *P. lutzii* database by PLGS (PepFrag 1 and PepFrag 2), variables modifications (VarMod), fragmentation occurred in ionization source (InSource), missed cleavage by trypsin occurred during digestion (Missed Cleavage) and Neutral loss H_2O and NH_3 corresponding, respectively, to water and ammonia precursor losses.

Table S1. List of primers used in RT-qPCR experiments.

Gene / Accession number [a]	Forward primer (5´-3´)	Reverse primer (5´-3´)
Tubulin / PAAG_03031	GAGCGATTCATTGGAGGGATT	ATCAGGGAAAACAGAGTAAGTC
Arginase / PAAG_01969	TCCGGAGGCCAGAGCA	TGACGGTGTAGCCTAGGTCT
Formamidase /PAAG_03333	GTTCTATCCCAATGCCGCAAA	GCGCCTGTTCCATTCAGCTA
γ-Glutamyl transpeptidase / PAAG_06130	ACTGGTGGGTTGGCTAGCG	GCGAATGGCAGGCTGCATG
Urease / PAAG_00954	GAGATATATGTTTGGGGCACG	ACCTCGACAATTACGCACCG

[a] Accession number of genes used in quantitative real time PCR from NCBI genome database (https://www.ncbi.nlm.nih.gov/genome/?term=paracoccidioides).

Table S2. *P. lutzii* up-regulated proteins under NCR conditions.

Accession Number [a]	Protein name [b]	Score [c]	Fold change [d]
**METABOLISM**			
**Amino acid metabolism**			
PAAG_02859	Adenosylhomocysteinase	1172 .15	*
PAAG_07327	Arginine biosynthesis bifunctional protein ArgJ	950.33	*
PAAG_08065	Aspartate-semialdehyde dehydrogenase	1046.73	*
PAAG_08163	Fumarylacetoacetase	3595.62	*
PAAG_00285	Imidazole glycerol phosphate synthase hisHF	912.15	*
PAAG_02644	Kynurenine-oxoglutarate transaminase	1567.78	*
PAAG_08100	O-acetylhomoserine (Thiol)-lyase	1026.41	*
PAAG_06096	Phospho-2-dehydro-3-deoxyheptonate aldolase	1614.21	*
PAAG_01302	S-methyl-5'-thioadenosine phosphorylase	783.05	*
PAAG_04443	Spermidine synthase	1061.64	*
PAAG_07003	Glutamine synthetase	1704.34	*
PAAG_03138	Alanine-glyoxylate aminotransferase	1352.65	*
PAAG_05328	3-isopropylmalate dehydrogenase	1317.29	*
PAAG_07467	Dipeptidyl peptidase 3	889.36	*
PAAG_06217	Acetylornithine aminotransferase	1067.15	*
PAAG_00691	Fumarylacetoacetate hydrolase domain-containing protein	904.53	1.336427477
PAAG_01568	Glycine cleavage system P protein	1341.16	1.336427477
PAAG_08512	Serine hydroxymethyltransferase	1230.06	1.363425117
PAAG_07036	Methylmalonate-semialdehyde dehydrogenase	1164.09	1.377127754
PAAG_06168	Peptidyl-prolyl cis-trans isomerase	2230.03	1.377127754
PAAG_07114	Argininosuccinate synthase	1279.04	1.390968147
PAAG_07760	Threonine synthase	1058.95	1.404947596
PAAG_01158	phenylpyruvate tautomerase	3328.09	1.404947596
PAAG_07813	Cysteine synthase	5107.64	1.491824707
PAAG_07412	Serine hydroxymethyltransferase	1177	1.50681778
PAAG_08718	Succinate-semialdehyde dehydrogenase	2972.25	1.616074385
PAAG_07605	Acetolactate synthase small subunit	2070.52	2.095935534
PAAG_01310	2-oxoisovalerate dehydrogenase subunit alpha	1504.79	2.1382762
PAAG_05417	Mitochondrial-processing peptidase subunit beta	1155.87	1.648721271
PAAG_05253	Delta-1-pyrroline-5-carboxylate dehydrogenase	1012.88	2.247907992
PAAG_03045	Aminomethyltransferase	2171.24	2.270499821
PAAG_06387	Homoisocitrate dehydrogenase	918.92	2.293318702
PAAG_07689	NADP-specific Glutamate dehydrogenase	867.37	2.316366916
PAAG_01974	Mitochondrial methylglutaconyl-CoA hydratase	2188.74	2.534509196
PAAG_01991	Homoserine dehydrogenase	1686.54	2.944679677
PAAG_06776	Peptide methionine sulfoxide reductase msrA	1511.01	7.463317276
PAAG_02217	Isochorismatase domain-containing protein	2112.56	3.287081395
**Nucleotide/nucleoside/nucleobase metabolism**			
PAAG_06906	Adenine phosphoribosyltransferase	785.27	*
PAAG_00433	Adenosine kinase	1005.07	*
PAAG_02354	Serine 3-dehydrogenase	1871.12	*
PAAG_05803	Inosine-5'-monophosphate dehydrogenase	834.36	1.584073998

PAAG_06643	Uracil phosphoribosyltransferase	2054.03	1.840431425
**C-compound and carbohydrate metabolism**			
PAAG_00545	Alpha-1.4 glucan phosphorylase/ glycon phosphorylase	878.38	*
PAAG_04478	Dienelactone hydrolase family protein	886.38	2.316366916
PAAG_06083	Dienelactone hydrolase family protein	1354.83	1.599994191
PAAG_04181	Sorbitol utilization protein SOU2	7006.95	2.054433269
PAAG_05780	2.5-diketo-D-gluconic acid reductase A	1502.34	4.349235265
PAAG_03516	Glucose-methanol-choline oxidoreductase	864.79	*
PAAG_01935	Formyl-coenzyme A transferase	958.18	*
PAAG_05780	D-xylose reductase	1502.34	4.349235265
**Metabolism of cofactors and vitamins**			
PAAG_08349	C-1-tetrahydrofolate synthase	754.22	*
PAAG_01324	Folic acid synthesis protein	5102.22	*
PAAG_01227	3-methyl-2-oxobutanoate hydroxymethyltransferase	1243.72	*
PAAG_08954	GTP cyclohydrolase	1070.03	*
PAAG_02980	Uncharacterized protein	848.69	*
PAAG_08856	Nicotinate-nucleotide pyrophosphorylase [carboxylating]	864.48	2.1382762
PAAG_07321	Pyridoxine biosynthesis protein PDX1	1226.82	2.829216906
PAAG_11728	Aminopeptidase	789.48	*
PAAG_00664	Aspartyl aminopeptidase	3021.46	*
PAAG_03719	Thimet oligopeptidase / metallopeptidase MepB	1103.4	*
PAAG_04205	Vacuolar aminopeptidase	1013.46	*
**Fatty acid biosynthesis and Lipid metabolism**			
PAAG_04489	Acyl-protein thioesterase	849.68	*
PAAG_07582	Uncharacterized protein	990.78	*
PAAG_06406	Fatty acid transporter protein	780.17	*
PAAG_07492	Ethanolamine-phosphate cytidylyltransferase	787.37	*
PAAG_01536	Trans-2-enoyl-CoA reductase	838.46	*
PAAG_07347	Phytanoyl-CoA dioxygenase family protein	780.3	*
PAAG_03631	12-oxophytodienoate reductase	4433.5	1.934792385
PAAG_06215	Hydroxymethylglutaryl-CoA lyase	859.44	2.509290432
PAAG_00953	NADH-cytochrome b5 reductase	876.55	3.490342957
**ENERGY**			
**Glycolysis and gluconeogenesis**			
PAAG_03243	Glucose-6-phosphate 1-epimerase	1845.01	*
PAAG_06526	Glucose-6-phosphate isomerase	3688.6	*
PAAG_06953	Short chain dehydrogenase/reductase family	803.6	*
PAAG_03910	Aldehyde dehydrogenase	3249.57	*
PAAG_02869	Phosphoglycerate kinase	3045.31	1.41906754
PAAG_00726	Pyruvate carboxylase	1774.85	1.768267039
PAAG_01995	Fructose-bisphosphate aldolase 1	3565.75	1.915540783
PAAG_00403	Alcohol dehydrogenase	1874.94	1.934792385
PAAG_05249	Aldehyde dehydrogenase	5071.08	2.054433269
PAAG_08911	Alcohol dehydrogenase	1047.18	2.1382762
PAAG_01015	Phosphotransferase /Hexokinase	1658.53	2.773194711
**Glyoxylate cycle**			

PAAG_06951	Isocitrate lyase	849.64	*
PAAG_04542	Malate synthase	1056.7	1.648721271
PAAG_04550	Citrate synthase	2432.44	11.35888284
**Tricarboxylic-acid pathway**			
PAAG_05150	ATP-citrate synthase subunit 1	1165.03	*
PAAG_05151	ATP-citrate-lyase	881.07	*
PAAG_00588	Fumarate hydratase	1503	1.323129814
PAAG_00053	Malate dehydrogenase	16362.69	1.648721271
PAAG_03330	Dihydrolipoyl dehydrogenase	4001.51	1.840431425
PAAG_02769	Dihydrolipoamide acetyltransferase	1091.95	*
PAAG_01556	FAD dependent oxidoreductase superfamily	784.56	*
**Electron transport and membrane-associated energy conservation**			
PAAG_06268	Cytochrome c	1392.83	*
PAAG_08551	ATP synthase subunit g	1369.04	*
PAAG_04570	ATP synthase subunit d. mitochondrial	951.57	1.616074385
PAAG_07921	cytochrome c oxidase assembly factor 6	2805.94	2.339646908
**Methylcitrate Cycle**			
PAAG_04559	2-methylcitrate dehydratase	4830.94	1.768267039
**Pentose phosphate pathway**			
PAAG_00633	Glucose-6-phosphate 1-dehydrogenase	824.2	*
PAAG_02633	Ribose-phosphate pyrophosphokinase	788.55	*
PAAG_01178	6-phosphogluconate dehydrogenase. decarboxylating	1053.33	1.476980773
PAAG_11415	Phosphoglucomutase	1020.36	1.599994191
**Oxidation of fatty acids**			
PAAG_05093	Succinyl-CoA:3-ketoacid-coenzyme A transferase	1329.64	*
PAAG_06224	Carnitine O-acetyltransferase	737.96	*
PAAG_00435	(R)-benzylsuccinyl-CoA dehydrogenase/ Acyl-CoA dehydrogenase	990.78	*
PAAG_06329	3-hydroxybutyryl-CoA dehydrogenase	3071.09	1.50681778
PAAG_02664	3-ketoacyl-CoA thiolase	2246.29	2.459603053
**CELL WALL HOMEOSTASIS**			
PAAG_04602	Mannosyl-oligosaccharide glucosidase	915.53	*
PAAG_07150	UDP-galactopyranose mutase	1202.01	*
PAAG_06817	UTP--glucose-1-phosphate uridylyltransferase	1160.54	1.349858824
**CELLULAR PROCESSES**			
PAAG_06192	Actin-related protein 2/3 complex subunit 4	1192.57	*
PAAG_07185	Actin-related protein 2/3 complex subunit 5	3374.47	*
PAAG_07702	ADP-ribosylation factor	1058.35	*
PAAG_05061	AhpC/TSA family protein	2253.84	*
PAAG_00875	ARP2/3 actin-organizing complex subunit Sop2	792.17	*
PAAG_02409	WD repeat domain phosphoinositide-interacting protein	1049.41	*
PAAG_01500	GTP-binding protein SAS1	2012.19	1.568312167
PAAG_05663	Arp2/3 complex 34 kDa subunit	1000.36	3.034358438
**SIGNALING AND CELLULAR PROCESSES**			
PAAG_08880	Cell division control protein	1112.17	*
PAAG_00004	Coronin	3410.12	*

PAAG_07958	Fimbrin	1705.51	*
PAAG_03551	Calcineurin subunit B	1512.49	*
PAAG_08247	Calmodulin	1990.66	1.404947596
PAAG_06751	DNA damage checkpoint protein rad24	15208.9	1.537257535
PAAG_00773	DNA damage checkpoint protein rad24	5441.85	1.750672504
PAAG_08028	GTP-binding protein ypt1	1856.47	1.915540783
**GENETIC INFORMATION PROCESSING**			
**Chromosome and associated proteins**			
PAAG_02619	Uncharacterized protein	982.12	*
PAAG_05476	ADP-ribosylation factor family protein	818.37	*
PAAG_12620	Uncharacterized protein	1005.73	*
PAAG_00106	Histone acetyltransferase type B catalytic subunit	1028.61	*
PAAG_07098	Histone H4	4752.12	1.336427477
PAAG_02820	Obg-like ATPase 1	1162.41	1.336427477
PAAG_08918	Histone H2B	840.43	1.41906754
PAAG_00126	Histone H4	4787.73	1.462284582
PAAG_08471	Histone H2A	1078.66	1.803988368
**Transcription machinery**			
PAAG_01062	Small nuclear ribonucleoprotein Sm D1	1765.8	*
PAAG_08223	Small nuclear ribonucleoprotein Sm D3	960.98	*
PAAG_02437	U2 small nuclear ribonucleoprotein B	884.55	*
PAAG_07785	116 kDa U5 small nuclear ribonucleoprotein component	767.68	*
PAAG_01597	Uncharacterized protein	1487.41	*
PAAG_02033	Mediator of RNA polymerase II transcription subunit 31	762.91	*
PAAG_06996	G-protein complex beta subunit CpcB	2558.17	1.896480852
PAAG_02537	Small nuclear ribonucleoprotein Sm D2	838.81	2.1382762
PAAG_07296	single-strand DNA binding protein family	7351.35	3.18993317
**Translation**			
PAAG_00339	Poly(A)+ RNA export protein	750.12	*
PAAG_00244	Polyadenylate-binding protein	904.88	*
PAAG_07707	Ribosomal protein	2034.46	*
PAAG_00783	Serine/threonine-protein phosphatase	1287.19	*
PAAG_08702	Seryl-tRNA synthetase	787.75	*
PAAG_05103	Threonyl-tRNA synthetase	1593.24	*
PAAG_07841	Uncharacterized protein	27243.5	*
PAAG_08611	Uncharacterized protein	847.38	*
PAAG_01050	60S ribosomal protein L10-B	1764.72	*
PAAG_00088	60S ribosomal protein L3	858.39	*
PAAG_00648	60S ribosomal protein L33-B	825.72	*
PAAG_03019	60S ribosomal protein L6	878.89	*
PAAG_00347	60S ribosomal protein L9-B	3215.52	*
PAAG_04743	Casein kinase II subunit alpha	965.73	*
PAAG_04662	Cleavage and polyadenylation specificity factor subunit 5	2944.7	*
PAAG_04193	Cysteinyl-tRNA synthetase	833.13	*
PAAG_12263	Eukaryotic translation initiation factor 3 subunit M	901.03	*
PAAG_06891	mRNA binding post-transcriptional regulator (Csx1)	1030.51	*

PAAG_03941	G4 quadruplex nucleic acid binding protein	717.6	*
PAAG_11057	Uncharacterized protein	813.7	*
PAAG_06792	60S ribosomal protein L34-B	866.27	1.41906754
PAAG_09096	Uncharacterized protein	2804.24	1.41906754
PAAG_05704	40S ribosomal protein S13-1	5253.87	1.433329435
PAAG_03816	40S ribosomal protein S4	1032.77	1.462284582
PAAG_03322	40S ribosomal protein S20	1112.85	1.462284582
PAAG_00430	60S ribosomal protein L2	5063.03	1.552707215
PAAG_06367	30S ribosomal protein S17P	2372.65	1.568312167
PAAG_00205	Uncharacterized protein	1028.5	1.665291179
PAAG_03556	Elongation factor 1-gamma 1	3103.08	1.716006899
PAAG_03028	Elongation factor 1-beta	1849.14	2.033991215
PAAG_03828	40S ribosomal protein S9	4875.23	2.054433269
PAAG_01347	Actin cytoskeleton protein (VIP1)	1443.21	1.50681778
PAAG_04814	Nucleic acid-binding protein	1326.46	1.87761057
PAAG_00689	ATP-dependent RNA helicase eIF4A	1078.94	2.203396474
PAAG_01834	60S ribosomal protein L16	1373.58	2.293318702
PAAG_09043	40S ribosomal protein S2	2065.17	2.435129616
PAAG_05778	40S ribosomal protein S19	2068	2.484322599
PAAG_07182	40S ribosomal protein S7	888.36	2.801065755
PAAG_01435	40S ribosomal protein S16	3546.19	3.095656485
PAAG_02889	Ribosomal protein L35	1607.76	3.034358438
**Mitochondrial biogenesis**			
PAAG_04458	Prohibitin-1	965.54	*
PAAG_00109	Mitochondrial intermembrane space translocase subunit Tim	1153.25	*
PAAG_09072	Mitochondrial nuclease	1445.23	*
PAAG_12425	Mitochondrial outer membrane protein porin	1052.61	*
PAAG_08620	ADP. ATP carrier protein	1253.36	*
PAAG_00797	Mitochondrial protein import protein MAS5	1654.77	*
**FOLDING. SORTING AND DEGRADATION**			
PAAG_00763	Proteasome component PUP3	3662.21	*
PAAG_03687	Proteasome endopeptidase complex	1372.89	*
PAAG_00071	Proteasome subunit alpha type	1236.49	*
PAAG_08418	Proteasome subunit beta	1172.59	*
PAAG_00770	26S protease regulatory subunit	881.91	*
PAAG_01926	Uncharacterized protein	945.7	*
PAAG_07037	Calnexin	1089.94	*
PAAG_07589	SNARE domain-containing protein	902.64	*
PAAG_08006	V-SNARE	1225.94	*
PAAG_00852	Proteasome endopeptidase complex	1219.14	2.585709628
PAAG_06536	Ubiquitin	1659.56	2.664456293
PAAG_07080	Ubiquitin	1676.59	2.915379653
PAAG_07802	Proteasome subunit alpha type	1641.06	2.944679677
PAAG_00782	Small COPII coat GTPase sar1	2428.83	3.596639623
PAAG_00866	Proteasome component C5	1149.46	3.78104355
PAAG_01941	Proteasome subunit alpha type	1048.74	3.78104355

**CELL RESCUE. DEFENSE AND VIRULENCE**			
PAAG_05716	Carbonic anhydrase	2849.07	*
PAAG_06947	Gamma-glutamyltranspeptidase	745.77	*
PAAG_05292	Glutathione reductase	2009.44	*
PAAG_06237	Urease accessory protein ureG	1325.73	*
PAAG_01465	Carbonic anhydrase	8067.02	1.840431425
PAAG_03333	Formamidase	957.69	2.484322599
**Detoxification**			
PAAG_02725	Superoxide dismutase Fe/Mn	3674.03	2.117000017
PAAG_03216	Mitochondrial peroxiredoxin PRX1	7829.13	1.648721271
PAAG_06175	Peroxisomal matrix protein	2592.75	1.69893226
PAAG_03931	Glutathione S-transferase Gst3	1523.77	2.1382762
PAAG_08859	Peroxisomal multifunctional enzyme	1347.9	1.390968147
PAAG_08931	Glutamate carboxypeptidase	827.03	2.316366916
PAAG_04164	Superoxide dismutase	1569.92	2.509290432
PAAG_02364	Thioredoxin	1639.01	3.095656485
PAAG_01368	Benzoate 4-monooxygenase cytochrome P450	800.11	*
**Stress response/Chaperones and folding catalysts**			
PAAG_07775	Heat shock protein SSB1	813.79	2.1382762
PAAG_02116	Hsp70	737.97	*
PAAG_11262	Hsp7-like protein	6986.88	*
PAAG_01262	Hsp70-like protein	4095.83	1.41906754
PAAG_06811	Heat shock protein STI1	1282.75	1.433329435
PAAG_07750	Heat shock protein Hsp88	4564.28	1.599994191
PAAG_03334	Peptidyl-prolyl cis-trans isomerase D	1423.35	1.616074385
PAAG_03292	Peroxidase	1335.5	1.323129814
PAAG_08260	Hsp90 co-chaperone Cdc37	1503.88	2.159766213
PAAG_05788	Peptidyl-prolyl cis-trans isomerase	1468.5	2.611696417
**HYPOTHETICAL PROTEINS**			
PAAG_00090	Uncharacterized protein	1514.25	*
PAAG_00471	Uncharacterized protein	1217.01	*
PAAG_00541	Uncharacterized protein	1601.72	*
PAAG_01045	Uncharacterized protein	3809.36	*
PAAG_01124	Uncharacterized protein	1449.93	*
PAAG_01231	Uncharacterized protein	740.54	*
PAAG_02154	Uncharacterized protein	2371.2	*
PAAG_02768	Uncharacterized protein	1079.22	*
PAAG_02985	Uncharacterized protein	890.65	*
PAAG_03092	Uncharacterized protein	1775.68	*
PAAG_03967	Uncharacterized protein	979.14	*
PAAG_04037	Uncharacterized protein	1046.63	*
PAAG_04358	Uncharacterized protein	900.14	*
PAAG_04647	Uncharacterized protein	933.51	*
PAAG_05388	Uncharacterized protein	796.82	*
PAAG_05550	Uncharacterized protein	1650.16	*
PAAG_07035	Uncharacterized protein	795.57	*

PAAG_07886	Uncharacterized protein	718.56	*
PAAG_07957	Uncharacterized protein	1951.39	*
PAAG_07989	Uncharacterized protein	1618.79	*
PAAG_11844	Uncharacterized protein	1414.51	*
PAAG_12508	Uncharacterized protein	1601.67	*
PAAG_02608	Uncharacterized protein	2025.05	*
PAAG_05041	Altered inheritance of mitochondria protein 24. mitochondrial	887.68	*
PAAG_03701	BAR domain-containing protein	868.29	*
PAAG_01875	Uncharacterized protein	940.14	*
PAAG_01135	Uncharacterized protein	877.51	*
PAAG_00743	Zinc metalloprotease	1067.74	*
PAAG_01399	NAD dependent epimerase/dehydratase family protein	784.47	*
PAAG_01717	Uncharacterized protein	768.17	*
PAAG_01928	Peroxisomal dehydratase	898.55	*
PAAG_05019	Uncharacterized protein	11596.59	1.896480852
PAAG_03421	Uncharacterized protein	1227.75	1.323129814
PAAG_07772	Uncharacterized protein	1237.8	1.404947596
PAAG_01854	Uncharacterized protein	1151.47	1.733253039
PAAG_05259	Uncharacterized protein	2698.97	2.181472203
PAAG_00335	Uncharacterized protein	1782.49	2.203396474
PAAG_00579	Uncharacterized protein	1167.2	2.225540955
PAAG_07262	Oxidoreductase	1372.88	2.459603053
PAAG_01321	Oxidoreductase 2-nitropropane dioxygenase family	1373.44	1.954237353

[a] Identification of differentially regulated proteins from *P. lutzii* genome database (https://www.ncbi.nlm.nih.gov/genome/?term=paracoccidioides) using ProteinLynx Global server v.3.0.2 (PLGS) (Water Corporation. Manchester. UK);

[b] Proteins annotation from *P. lutzii* genome database or by homology in BlastP in NCBI database (https://blast.ncbi.nlm.nih.gov/Blast.cgi?PROGRAM=blastp&PAGE_TYPE=BlastSearch&LINK_LOC=blasthome);

[c] PLGS score is the result of different mathematical models for peptide and fragment assignment prediction;

[d] Fold-change values were obtained by dividing the values of protein abundance (in fmol) from *P. lutzii* yeast cells during nitrogen limiting conditions by cultured in Yeast Nitrogen Base (YNB) without amino acids and ammonium sulfate (Sigma-Aldrich®) supplemented with 10mM glutamine and 10mM proline nitrogen sources. Proteins with a minimum fold-change of 30% were considered to be regulated;

[*] Proteins identified only in *P. lutzii* cultured in 10 mM proline nitrogen source.

Table S3. Down-regulated proteins in *P. lutzii* under NCR conditions.

Accession number [a]	Protein name [b]	Score [c]	Fold change [d]
**GENETIC INFORMATION PROCESSING**			
**Translation**			
PAAG_01433	40S ribosomal protein S14	895.21	*
PAAG_05946	40S ribosomal protein S27	1098.08	*
PAAG_05117	Aspartyl-tRNA synthetase	926.51	*
PAAG_04511	ATP-dependent RNA helicase SUB2	1148.14	*
PAAG_04396	Casein kinase II subunit beta	885.2	*
PAAG_01050	Cytosolic large ribosomal subunit protein L30	2255.3	*
PAAG_08172	Lysine--tRNA ligase	846.52	*
PAAG_04691	Uncharacterized protein	1136.13	*
PAAG_04958	Eukaryotic translation initiation factor 6	2682.7	0.522045789
PADG_06525	40S ribosomal protein S1	1329.16	0.301194198
PAAG_06320	60S ribosomal protein L13	2011.37	0.319819026
PAAG_01785	40S ribosomal protein S3	1751.42	0.343008499
PAAG_05805	40S ribosomal protein S21	2125.76	0.49658531
PAAG_05484	40S ribosomal protein S5	1524.23	0.548811623
PAAG_08888	60S ribosomal protein L4-A	2081.11	0.58274824
PAAG_06487	60S ribosomal protein L7-C	902.78	0.625002269
PAAG_11418	Elongation factor 1-alpha	2254.68	0.63128364
PAAG_00801	60S acidic ribosomal protein P0	1991.82	0.644036423
PAAG_04998	60S ribosomal protein L8-B	2445.15	0.677056884
PAAG_02921	Elongation factor Tu	1359.54	*
PAAG_04571	Nascent polypeptide-associated complex subunit alpha	2494.82	0.151071811
**Transcription machinery**			
PAAG_00727	Mitosis protein dim1	1375.32	*
PAAG_04496	Nascent polypeptide-associated complex subunit beta	1155.68	0.26184566
PAAG_01097	Poly(RC)-binding protein	879.46	*
**FOLDING. SORTING AND DEGRADATION**			
PAAG_02255	mRNA decapping hydrolase	1118.01	*
PAAG_12063	Protein transporter SEC23	1042.56	0.49658531
PAAG_02907	26S proteasome non-ATPase regulatory subunit 10	1008.82	*
PAAG_08141	Proteasome subunit alpha type	1225.3	0.458406024
**SIGNALING AND CELLULAR PROCESSES**			
PAAG_00317	Septin 4	1150.03	*
PAAG_04042	Sporulation-regulated protein	885.78	*
PAAG_03309	Suaprga1	1255.1	*
PAAG_08058	cofilin	967.66	*
PAAG_08093	GTP-binding protein ypt3	1335.57	*
PAAG_03161	GTP-binding protein ypt5	1680.55	*
PAAG_01602	Ras-like GTP-binding protein	1045.44	*
PAAG_03669	Phosphatidylglycerol/phosphatidylinositol transfer protein	1520.4	*
PAAG_06344	Rab GDP dissociation inhibitor	1394.62	*
PAAG_03532	Actin	2121.1	0.527292432

PAAG_01647	Tubulin alpha chain	2875.62	0.644036423
**ENERGY**			
**Glycolysis and gluconeogenesis**			
PAAG_02050	Pyruvate decarboxylase	1354.24	*
PAAG_01534	Pyruvate dehydrogenase E1 component subunit beta	1195.73	*
PAAG_11035	Acetyltransferase component of pyruvate dehydrogenase complex	2275.17	0.301194198
PADG_02411	Glyceraldehyde-3-phosphate dehydrogenase	4028.65	0.571209062
PAAG_06473	Mannitol-1-phosphate 5-dehydrogenase	892.82	0.670320042
**Tricarboxylic-acid pathway**			
PAAG_07729	Isocitrate dehydrogenase [NAD] subunit. mitochondrial	1111.89	*
PAAG_05415	Succinate dehydrogenase [ubiquinone] cytochrome b small subunit	910.29	*
PAAG_00417	Succinate--CoA ligase [ADP-forming] subunit alpha. mitochondrial	892.95	*
**Oxidation of fatty acids**			
PAAG_07786	Acetyl-CoA acetyltransferase	1673.49	*
PAAG_05454	Acyl-CoA dehydrogenase	793.59	0.114177608
**Electron transport and membrane-associated energy conservation**			
PAAG_04820	ATP synthase subunit alpha	1259.84	*
**METABOLISM**			
**Amino acid metabolism**			
PAAG_03978	3-hydroxyisobutyrate dehydrogenase	1672.59	*
PAAG_08405	Cystathionine beta-lyase	1000.4	*
**Fatty acid biosynthesis and Lipid metabolism**			
PAAG_03024	3-ketodihydrosphingosine reductase tsc10	1072.59	*
PAAG_06309	Enoyl-CoA hydratase	987.56	0.075020047
**Metabolism of cofactors and vitamins**			
PAAG_02071	Glutamyl-tRNA synthetase	879.68	*
PAAG_03345	Phosphotyrosine protein phosphatase	6980.18	0.436049294
PAAG_12076	NAD(P)H:quinone oxidoreductase. type IV	12494.06	0.406569669
**SIGNALING AND CELLULAR PROCESSES**			
**Stress response/Chaperones and folding catalysts**			
PAAG_05142	10 kDa heat shock protein. mitochondrial	24841.12	0.697676316
PAAG_01778	Peptidyl-prolyl cis-trans isomerase	1508.67	0.225372653
PAAG_05679	Heat shock protein	5649	0.683861412
**Detoxification**			
PAAG_04424	Glutathione peroxidase	1304.27	0.571209062
**HYPOTHETICAL PROTEINS**			
PAAG_06462	Diazepam-binding inhibitor	1748.96	*
PAAG_00297	Uncharacterized protein	1243.69	*
PAAG_00340	Uncharacterized protein	1222.61	*
PAAG_00928	Uncharacterized protein	1505.13	*
PAAG_05564	Uncharacterized protein	1345.97	*
PAAG_07405	Uncharacterized protein	2187.11	*
PAAG_11812	Uncharacterized protein	946.94	*

PAAG_01567	NTP_transf_9 domain-containing protein	1480.79	*

[a] Identification of differentially regulated proteins from *P. lutzii* genome database (https://www.ncbi.nlm.nih.gov/genome/?term=paracoccidioides) using ProteinLynx Global server v.3.0.2 (PLGS) (Water Corporation. Manchester. UK);

[b] Proteins annotation from *P. lutzii* genome database or by homology in BlastP in NCBI database (https://blast.ncbi.nlm.nih.gov/Blast.cgi?PROGRAM=blastp&PAGE_TYPE=BlastSearch&LINK_LOC=blasthome);

[c] PLGS score is the result of different mathematical models for peptide and fragment assignment prediction;

[d] Fold-change values were obtained by dividing the values of protein abundance (in fmol) from *P. lutzii* yeast cells during nitrogen limiting conditions by cultured in Yeast Nitrogen Base (YNB) without amino acids and ammonium sulfate (Sigma-Aldrich®) supplemented with 10mM glutamine and 10mM proline nitrogen sources. Proteins with a minimum fold-change of 30% were considered to be regulated;

[*] Proteins identified only in *P. lutzii* cultured in 10 mM proline nitrogen source.

Table S4. Amino acid biosynthesis and degradation of the up-regulated proteins under NCR-proline conditions.

**BIOSYNTHESIS**		
**Accession number [1]**	**Protein name**	**Metabolic pathway ***
PAAG_02859	Adenosylhomocysteinase	Cysteine and methionine metabolism
PAAG_07327	Arginine biosynthesis bifunctional protein ArgJ	Arginine biosynthesis
PAAG_08065	Aspartate-semialdehyde dehydrogenase	Glycine, serine and threonine metabolism
PAAG_00285	Imidazole glycerol phosphate synthase hisHF	Histidine metabolism
PAAG_06096	Phospho-2-dehydro-3-deoxyheptonate aldolase	Phenylalanine, tyrosine and tryptophan biosynthesis
PAAG_07003	Glutamine synthetase	Arginine biosynthesis
PAAG_05328	3-isopropylmalate dehydrogenase	Valine, leucine and isoleucine biosynthesis
PAAG_06217	Acetylornithine aminotransferase	Arginine biosynthesis
PAAG_07114	Argininosuccinate synthase	Arginine biosynthesis
PAAG_07760	Threonine synthase	Glycine, serine and threonine metabolism
PAAG_07813	Cysteine synthase	Cysteine and methionine metabolism
PAAG_07605	Acetolactate synthase small subunit	Valine, leucine and isoleucine biosynthesis
PAAG_06387	Homoisocitrate dehydrogenase	Lysine biosynthesis
PAAG_07689	NADP-specific Glutamate dehydrogenase	Arginine biosynthesis
PAAG_01991	Homoserine dehydrogenase	Glycine, serine and threonine metabolism
PAAG_02217	Isochorismatase domain-containing protein	Phenylalanine, tyrosine and tryptophan biosynthesis
**DEGRADATION**		
**Access number [1]**	**Protein name**	**Metabolic pathway ***
PAAG_08163	Fumarylacetoacetase	Tyrosine degradation
PAAG_02644	Kynurenine-oxoglutarate transaminase	Tyrosine degradation
PAAG_08100	O-acetylhomoserine (Thiol)-lyase	Cysteine and methionine metabolism
PAAG_01302	S-methyl-5'-thioadenosine phosphorylase	Cysteine and methionine metabolism
PAAG_04443	Spermidine synthase	Cysteine and methionine metabolism
PAAG_03138	Alanine-glyoxylate aminotransferase	Alanine, aspartate and glutamate metabolism
PAAG_00691	Fumarylacetoacetate hydrolase domain-containing protein	Tyrosine metabolism
PAAG_01568	Glycine cleavage system P protein	Glycine, serine and threonine metabolism
PAAG_08512	Serine hydroxymethyltransferase	Glycine, serine and threonine metabolism
PAAG_07036	Methylmalonate-semialdehyde dehydrogenase	Valine, leucine and isoleucine degradation
PAAG_01158	phenylpyruvate tautomerase	Tyrosine metabolism
PAAG_07412	Serine hydroxymethyltransferase	Glycine, serine and threonine metabolism
PAAG_08718	Succinate-semialdehyde dehydrogenase	Alanine, aspartate and glutamate metabolism
PAAG_01310	2-oxoisovalerate dehydrogenase subunit alpha	Valine, leucine and isoleucine degradation
PAAG_05253	Delta-1-pyrroline-5-carboxylate dehydrogenase	Alanine, aspartate and glutamate metabolism
PAAG_03045	Aminomethyltransferase	Glycine, serine and threonine metabolism
PAAG_01974	Mitochondrial methylglutaconyl-CoA hydratase	Valine, leucine and isoleucine degradation

[1] Accession number and protein available in Uniprot databases (https://www.uniprot.org/);

* Metabolic pathway available in Kegg pathway database (https://www.genome.jp/kegg/pathway.html).

Table S5. Proteins up-regulated in *P. lutzii* related with adherence under NCR-proline conditions.

Accession number [1]	Protein name	Faapred [2]
PAAG_08065	Aspartate-semialdehyde dehydrogenase	0.78097396
PAAG_02769	Dihydrolipoamide acetyltransferase component of pyruvate dehydrogenase complex	0.7962463
PAAG_00285	Imidazole glycerol phosphate synthase hisHF	0.74109549
PAAG_08100	O-acetylhomoserine (Thiol)-lyase	0.63380869
PAAG_06217	Acetylornithine aminotransferase	0.7257679
PAAG_07605	Acetolactate synthase small subunit	0.03256339
PAAG_03045	Aminomethyltransferase	0.47874841
PAAG_06906	Adenine phosphoribosyltransferase	0.75626885
PAAG_02354	Serine 3-dehydrogenase	0.54436313
PAAG_06643	Uracil phosphoribosyltransferase	0.54851004
PAAG_08349	C-1-tetrahydrofolate synthase	0.72916011
PAAG_08856	Nicotinate-nucleotide pyrophosphorylase [carboxylating]	0.55258796
PAAG_06215	Hydroxymethylglutaryl-CoA lyase	0.68521882
PAAG_00403	Alcohol dehydrogenase	0.54396443
PAAG_07921	cytochrome c oxidase assembly factor 6	0.29603461
PAAG_02664	3-ketoacyl-CoA thiolase	0.73062744
PAAG_07185	Actin-related protein 2/3 complex subunit 5	0.67173705
PAAG_00875	ARP2/3 actin-organizing complex subunit Sop2	0.6649768
PAAG_02033	Mediator of RNA polymerase II transcription subunit 31	0.30093731
PAAG_06996	G-protein complex beta subunit CpcB	0.29293222
PAAG_07296	single-strand DNA binding protein family	0.41982781
PAAG_03941	G4 quadruplex nucleic acid binding protein	0.62700526
PAAG_06792	60S ribosomal protein L34-B	0.31310686
PAAG_09096	Uncharacterized protein	0.45667675
PAAG_01347	Actin cytoskeleton protein (VIP1)	0.58667185
PAAG_07182	40S ribosomal protein S7	0.72150878
PAAG_01435	40S ribosomal protein S16	0.080352837
PAAG_09072	Mitochondrial nuclease	0.2554477
PAAG_08418	Proteasome subunit beta	0.35173182
PAAG_07037	Calnexin	0.56053173
PAAG_05716	Carbonic anhydrase	0.6073389
PAAG_06237	Urease accessory protein ureG	0.70113207
PAAG_02364	Thioredoxin	0.72402533
PAAG_03292	Peroxidase	0.6156496
PAAG_00090	Uncharacterized protein	0.2257914
PAAG_00541	Uncharacterized protein	0.037364734
PAAG_01124	Uncharacterized protein	0.39361832
PAAG_02154	Uncharacterized protein	0.43847169
PAAG_02768	Uncharacterized protein	0.47654572
PAAG_02985	Uncharacterized protein	0.74640492
PAAG_03967	Uncharacterized protein	0.023074998
PAAG_05388	Uncharacterized protein	0.77153754
PAAG_05550	Uncharacterized protein	0.64984866

PAAG_07035	Uncharacterized protein	0.44492444
PAAG_07886	Uncharacterized protein	0.4090276
PAAG_07957	Uncharacterized protein	0.37538801
PAAG_02608	Uncharacterized protein	0.025191829
PAAG_05041	Altered inheritance of mitochondria protein 24, mitochondrial	0.54592048
PAAG_01399	NAD dependent epimerase/dehydratase family protein	0.046511185
PAAG_00335	Uncharacterized protein	0.75377236
PAAG_00579	Uncharacterized protein	0.23839148
PAAG_01556	FAD dependent oxidoreductase superfamily	0.58681603
PAAG_08954	GTP cyclohydrolase	0.70326017
PAAG_07262	Oxidoreductase	0.58907821
PAAG_01321	Oxidoreductase 2-nitropropane dioxygenase family	0.42844503

[1] Accession numbers was obtained in NCBI database (https://www.ncbi.nlm.nih.gov/);

[2] Prediction of adhesins performed in FaaPred database (http://bioinfo.icgeb.res.in/faap/svmtool1.php).
